# Les kystes hydatiques du foie rompus dans les voies biliaires: à propos de 120 cas

**Published:** 2011-11-22

**Authors:** Mountassir Moujahid, Mohamed Tarik Tajdine

**Affiliations:** 1 Service de chirurgie générale, Hôpital militaire Avicenne, Marrakech, Maroc

**Keywords:** Kyste hydatique, foie, voies biliaires, diagnostic, traitement

## Abstract

Etude rétrospective rapportant une série de kystes hydatiques rompus dans les voies biliaires colligés dans le service de chirurgie de l'hôpital militaire Avicenne à Marrakech. Entre 1990 à 2008, sur 536 kystes hydatiques du foie opérés dans le service, 120 étaient compliqués de rupture dans les voies biliaires soit 22,38%. Il y avait 82hommes et 38 femmes. L’âge moyen était de 35 ans avec des extrêmes allant de 10 à 60 ans. La clinique était dominée par la crise d'angiocholite ou une douleur du flanc droit. L'ictère était isolé dans huit cas. La fistule biliokystique était latente dans plus de 50% des cas. Le traitement a consisté en une résection du dôme saillant dans103cas (85,84%), une périkystectomie chez 11 malades (9,16%) et une lobectomie gauche dans six cas (5%). Le traitement de la fistule bilio kystique a consisté en une suture chez 36malades et un drainage bipolaire dans 25 cas, La déconnexion kysto-biliaire ou cholédocotomie trans hépatico kystique selon Perdomo était pratiquée dans 49cas et une anastomose bilio-digestive cholédoco-duodénale dans 10 cas. La durée moyenne d'hospitalisation était de 20jours. Nous déplorons deux décès par choc septique et un troisième par encéphalopathie secondaire à une cirrhose biliaire. La morbidité était représentée par huit abcès sous phrénique, douze fistules biliaires prolongées et deux occlusions intestinales. Les kystes hydatiques rompus dans les voies biliaires représentent la complication la plus grave de cette pathologie bénigne. Le traitement repose sur des méthodes radicales qui sont d'une efficacité reconnue, mais de réalisation dangereuse et les méthodes conservatrices, en particulier la déconnexion kysto-biliaire qui est une méthode simple et qui donne de bons résultats à court et à long terme.

## Introduction

Le kyste hydatique du foie est une affection cosmopolite qui vit en état endémique dans les pays en voie de développement et qui représente un véritable problème de santé publique dans notre pays. C'est une maladie bénigne mais dont la plus fréquente et grave complication est représentée par la fistulisation du kyste dans les voies biliaires. Nous rapportons une étude rétrospective de 120 cas de kystes hydatiques du foie rompus dans les voies biliaires colligés dans le service de chirurgie générale de l'hôpital militaire Avicenne à Marrakech.

## Observations

Entre 1990 et 2008, sur 536 kystes hydatiques du foie opérés dans le service 120 cas étaient compliqués de rupture dans la voie biliaire (22,38%), il y avait 82 hommes et 38 femmes. L’âge moyen de nos patients est de 35 ans avec des extrêmes allant de 10ans à 60 ans. La notion de contact avec les chiens était rapportée dans plus de la moitié des cas. La symptomatologie clinique était dominée par la crise d'angiocholite, parfois par l'apparition d'une douleur de l'hypochondre droit ou une hépatomégalie fébrile. L'ictère était isolé dans huit cas (Tableau 1).

**Tableau 1:** répartition des symptômes cliniques chez 120 opérés pour kystes hydatiques du foie compliqués de rupture dans la voie biliaire dans le service de chirurgie générale de l'Hôpital Militaire Avicenne à Marrakech, Maroc, de 1990 and 2008

Symptômes	Fréquence (%)
Douleur de l'hypochondre droit	38(31,66%)
Angiocholite	47 (39,16%)
Ictère isolé	8(6,6%)
Hépatomégalie fébrile	27(22,5%)

L'imagerie médicale a permis de poser le diagnostic de kyste hydatique hépatique dans tous les cas. Il y avait 64 kystes uniques (45 à droite et 19 à gauche), Les kystes étaient multiples chez 40 patients variant entre deux à cinq par patients, la taille variait de 5 à10 cm. Le matériel hydatique a été détecté également au niveau de la voie biliaire principale dans 64 cas. Le scanner abdominal a été demandé seulement dans 30 cas devant l'aspect pseudo tumoral à l’échographie ce qui a permis de découvrir d'autres localisations hydatiques chez 11 patients: splénique (n= 3), péritonéale (n= 3), diaphragmatique (n=2), pulmonaire (n=2) et un cas au niveau du psoas.

La sérologie hydatique réalisée chez 20 patients était positive dans douze cas.

L'incision était une sous costale dans 63 cas, bisous costale dans 34 cas, médiane dans 20 cas et pat thoraco-phréno-laparotomie dans trois cas. Après protection par des champs imbibés d'eau oxygénée, le kyste était ponctionné avec un gros trocart, le liquide de ponction était teinté de bile; la fistule biliaire était évidente dans 100% des cas. Le parasite est stérilisé par de l'eau oxygénée à dix volumes, le traitement du kyste a été réalisé par une résection du dôme saillant dans 103 cas (85,84%), une périkystectomie dans 11 cas (9,16%) avec ligature du canal biliaire dans cinq cas et une hépatectomie gauche dans six cas (5%) (Tableau 2).

**Tableau 2:** traitement de la cavité résiduelle chez 120 opérés pour kystes hydatiques du foie compliqués de rupture dans la voie biliaire dans le service de chirurgie générale de l'Hôpital Militaire Avicenne à Marrakech, Maroc, de 1990 and 2008

Nature du traitement	Nombre de cas (%)
Résection du dôme saillant	103 (85,84%)
Périkystectomie	11(9,16%)
Hépatectomie gauche	6 (5%)

Nous avons réalisés une cholangiographie per opératoire chez 85 malades par voie transcystique après une cholécystectomie ou par ponction directe de la voie biliaire principale ce qui a permis de visualiser du matériel hydatique chez 64 malades et de visualiser une ou plusieurs fistules.

La désobstruction de la voie biliaire principale (VBP) a été réalisée après ouverture de la VBP chez 64 patients avec des pinces à calcul et des grands lavages avec du sérum salé hypertonique. La VBP a été ensuite drainée par un drain de Kher. Les fistules biliaires intra kystiques ont été repérées grâce à une exploration minutieuse de la cavité kystique restante, aidée au besoin par une épreuve au bleu de méthylène. Le traitement a été réalisé soit par une suture directe simple par des points en x au vicryl, associé à un drainage de la cavité kystique par des drains tubulés ou perforés en simple siphonage chez 36 malades, soit par un drainage bipolaire par un drain de Kher ou Pedinilli et drainage de la cavité résiduelle après suture de l'orifice fistuleux dans 25 cas.

La déconnexion kysto-biliaire ou cholédocotomie trans-hépatico kystique selon Perdomo était pratiquée dans 49cas et une anastomose bilio- digestive dans dix cas. L'intervention était complétée par le traitement de la cavité résiduelle par un capitonnage chez 40 patients et par une épiplooplastie chez 30 malades. Les autres kystes associés ont été également traités par trois splénectomies et une omenectomie chez deux patients.

Nous déplorons deux décès post opératoire suite à un état de choc, et un autre par une encéphalopathie secondaire à une cirrhose biliaire. La morbidité était marquée par huit abcès sous phrénique traités dans six cas par une ponction trans-pariétale et dans deux cas par une reprise chirurgicale, douze fistules biliaires externes prolongées qui ont fini par se tarir, deux occlusions intestinales, une pleurésie enkystée et quatre abcès de la paroi. La durée moyenne d'hospitalisation était de 20 jours avec des extrêmes allant de dix à quarante jours (Tableau 3).

**Tableau 3:** récapitulatif des résultats en fonction du traitement de la fistule bilio-kystique chez 120 opérés pour kystes hydatiques du foie compliqués de rupture dans la voie biliaire dans le service de chirurgie générale de l'Hôpital Militaire Avicenne à Marrakech, Maroc, de 1990 and 2008

Traitement	Nombre (%)	Séjour moyen (jours)	Morbidité Cas (%)	Mortalité Décès (%)
Suture simple	36(30%)	40	20 (16,66%)	1 (0,83%)
Drainage bipolaire	25(20,83%)	30	7 (5,83%)	2 (1,66%)
Déconnexion kysto-biliaire	49(40,83%)	28	0	0
Anastomose cholédoco-duodénale	10(8,33%)	10	0	0

## Discussion

La rupture des KHF dans les voies biliaires est une complication fréquente et grave pour cette affection bénigne (17 à 44%) des kystes hydatiques opérés [1]. Dans notre série elle est de 22,38%. Elle semble fréquente au niveau des kystes hydatiques du foie droit [2].

La symptomatologie clinique est dominée surtout par l'angiocholite [2,3], mais les formes latentes sont les plus fréquentes dans notre série impliquant la recherche systématique de la fistule biliaire au cours de toute chirurgie de kyste hydatique du foie.

L'imagerie médicale a permis de poser le diagnostic dans tous les cas. La tomodensitométrie permet de préciser les rapports exacts du kyste avec les pédicules vasculo-biliaires [4,5], permet de visualiser les débris hydatiques dans la VBP. La cholangio-IRM constitue la technique de choix dans l'exploration des voies biliaires [4,6,7]; elle n'a pas été réalisée dans notre série.

Le traitement est chirurgical, après stérilisation du parasite, Les méthodes radicales sont la périkystectomie totale et la résection hépatique qui paraissent disproportionnées pour une affection bénigne et surtout sévissant dans un pays d'endémie [4,8].

La résection du dôme saillant est une méthode simple, rapide, peu hémorragique mais source d'une morbidité assez importante [3,7,9].

La suture simple associée ou non à un drainage est une méthode simple mais avec un risque élevé de fuites biliaires prolongées à l'origine d'un long séjour hospitalier.

La déconnexion kysto biliaire nous apparaît comme la méthode de choix, cette technique nous a permis de résoudre le problème de la fistule bilio kystique [2,6,10,11].

C'est la méthode qui a été la plus fréquemment pratiquée dans notre série 41 % avec une morbidité postopératoire nulle et un séjour moyen de 28 jours. C'est une méthode qui nous a donné une grande satisfaction.

La désobstruction de la voie biliaire principale se fait par cholédocotomie et drainage par un drain de Kher.

## Conclusion

La rupture des kystes hydatiques dans les voies biliaires est une complication fréquente et grave .Le traitement est chirurgical qui repose sur des méthodes radicales d'efficacité reconnue mais qui paraissent disproportionnées pour cette affection généralement bénigne et des méthodes conservatrices dont la déconnexion kysto-biliaires reste la méthode de choix.
